# Preparation of Photoirradiation Molecular Imprinting Polymer for Selective Separation of Branched Cyclodextrins

**DOI:** 10.3390/molecules22020288

**Published:** 2017-02-21

**Authors:** Haoran Fan, Jinpeng Wang, Qingran Meng, Xueming Xu, Tianming Fan, Zhengyu Jin

**Affiliations:** 1 The State Key Laboratory of Food Science and Technology, Jiangnan University, 1800 Lihu Road, Wuxi 214122, China; fanhaoran0622@163.com (H.F.); jpwang1984@jiangnan.edu.cn (J.W.); mengqingranxzjscn@163.com (Q.M.); xmxubest@126.com (X.X.); 2 School of Food Science and Technology, Jiangnan University, 1800 Lihu Road, Wuxi 214122, China; 3 Synergetic Innovation Center of Food Safety and Nutrition, Jiangnan University, 1800 Lihu Road, Wuxi 214122, China; 4 Muyang Group Co., Ltd., Muyang Road, Yangzhou 225127, China; wangjinpeng1984@126.com

**Keywords:** molecular imprinting polymer, branched cyclodextrins, azobenzene derivatives, photoirradiation

## Abstract

In the present study, photoirradiation molecularly imprinted polymer (MIP) with azobenzene was used as a functional monomer for the selective separation of the branched cyclodextrins. The functional monomer 4-methacryloyloxy azobenzene (MAA) and the molecular template 6-*O*-α-d-maltosyl-β-cyclodextrin (G2-β-CD) were implemented for the molecular imprinting. The core-shell structure of photoirradiation MIP was visualized by the transmission electron microscopy (TEM). With Fourier transform infrared spectroscopy (FTIR) and thermogravimetric analysis (TGA), we identified that G2-β-CD was imprinted into the polymer and removed from the MIP. The binding association constant (*K_a_*) and the maximum number of the binding site (*N*_max_) were 1.72 × 10^4^ M^−1^ and 7.93 μmol·g^−1^ MIP, respectively. With alternate irradiation at 365 and 440 nm light, the prepared MIP reversibly released and rebound to the G2-β-CD, which resulted in the nearly zero amount of G2-β-CD in the solution. The HPLC results indicated that the purity of G2-β-CD could reach 90.8% after going through MIP. The main finding of our study was that the photoirradiation of MIP was an easy and effective method for the selective separation of the branched cyclodextrins.

## 1. Introduction

Branched cyclodextrins (CDs), in which one or two primary hydroxyl groups of CD are replaced by mono- or di-saccharides through the α-1,6 glycosidic bond, present advantages over the parent cyclodextrins (α-CD, β-CD, and γ-CD), such as higher bioadaptability, higher solubility, and lower hemolytic activity [1,2,3]. Generally, the branched CDs are produced either by the action of cyclodextrin glycosyl transferase (CGTase) on amylopectin, or by the condensation of the saccharide chains (glucose (G1), maltose (G2), and other saccharides) and CDs with such debranching enzymes as pullulanase or isoamylase [4], and branched CDs, unbranched CDs, and acyclic dextrins are formed in the system [5]. Special chromatographic methods are used for separating and purifying branched CDs from acyclic dextrins yet are impractical for the commercial operations. For instance, Koizumi et al. isolated three branched CDs by high-performance liquid chromatography (HPLC) from the mother liquors of a large-scale preparation of unbranched CDs with *Bacillus ohbensis* cyclomaltodextrin glucanotransferase [2]. Ammeraal et al. developed a method for separating and purifying branched β-CD from an aqueous solution [5]. In this method, a β-CD complexant which was selected from the group consisting of *p*-xylene and toluene was added to a starting solution, the precipitate form was recovered and then dissolved in water. A second dose of the complexant was added to the solution, and a branched β-CD was recovered from the resulting liquor. The separation and purification of branched CDs from the solution is relatively difficult, complicated, and unsuitable on a large scale [6]. The easy and effective method for purifying and separating branched CDs from the mother liquors needs to be developed.

Molecular imprinting is a useful tool for preparing specific molecular recognition materials, which have tailor-made binding sites for the template molecules [7]. In molecularly imprinted polymer (MIP) syntheses, the functional monomer and the template molecule are assembled through either covalent or non-covalent interactions (such as ionic interactions, hydrogen bonds, metal-ion chelating interactions, and hydrophobic interactions) and co-polymerized with a crosslinking monomer [8,9]. After polymerization, the template molecule is removed from the polymer, yielding an MIP with specific selective binding cavities that are complementary to the size, shape, and functionality of the template molecule [10,11]. The MIP has many appealing characteristics such as physical robustness, thermal stability, and desired selectivity as well as low cost and easy preparation [12,13]. These physicochemical characteristics make MIP the promising candidates for applications in separation [14], drug-delivery [15], and chemical sensing [16]. Recently, much research attention was attracted to the MIP responsivity to stimuli, such as temperature, pH, ionic and solvent compositions, concentration of specific chemical or biochemical species, electric fields, and light irradiation [17]. In particular, light is a clean stimulus and its irradiation can be manipulated rapidly and precisely. Compared with other MIP, a template molecule uptaken by a photoirradiation MIP could be efficiently and conveniently released under appropriate light irradiation instead of eluting the template molecule in a solution for a longer time. Gong et al. designed a photoirradiation functional monomer 4-((4-methacryloyloxy)phenylazo)benzoic acid for preparing photoirradiation MIP with the aim of uptaking and releasing caffeine [18]. Gong et al. reported that photoirradiation of molecularly imprinted hydrogels is capable of releasing and uptaking pharmaceuticals in the aqueous media, where 4-((4-methacryloyloxy)phenylazo)benzenesulfonic acid is used as a functional monomer [19]. Gomy et al. reported a photoirradiation of MIP containing azo monomer di (ureidoethylenemethacrylate)azobenzene and their binding properties were investigated [20]. However, there is no report about demonstrating a photoirradiation of MIP for selective separation of branched CDs. In the present study, the template molecule 6-*O*-α-d-maltosyl-β-cyclodextrin (G2-β-CD) and the functional monomer 4-methacryloyloxy azobenzene (MAA) were used for molecular imprinting.

Among the branched CDs, 6-*O*-α-d-maltosyl-β-cyclodextrin (G2-β-CD) is generally the most accessible and useful CD because G2-β-CD has much higher solubilizing ability in water and aqueous methanol solution, higher resistance of the enzymatic degradation, lower local tissue irritancy, and weaker hemolytic activity on human erythrocytes than its parent β-CD [21]. G2-β-CD possesses a hydrophilic outer surface and hydrophobic internal cavity [22]. This structure is able to form the inclusion complexes with the azobenzene based on the *cis-trans* photoisomerization of azobenzene. Therefore, it is possible that the preparation of the photoirradiation MIP could be implemented for the selective separation of the branched cyclodextrins. The G2-β-CD is synthesized either by grafting maltose through the reverse action with isoamylase or pullulanase in a reaction mixture containing of β-CD and maltose, or by transferring maltosyfluoride to CDs via pullulanase [23]. To our knowledge, no previous study has demonstrated the preparation of the photoirradiation of MIP for the selective separation of branched CDs by using azobenzene and its derivatives as the functional monomer. In the current study, we prepared photoirradiation of MIP by using 4-methacryloyloxy azobenzene (MAA) as the functional monomer to separate G2-β-CD from the maltose. We assumed this approach is a simple and efficient separation process for G2-β-CD.

## 2. Results and Discussion

### 2.1. Characterization of the 4-(Phenyldiazenyl)phenol and MAA

It has been well established that a suitable functional monomer is crucial for the successful preparation of the MIP for selective separation of the branched cyclodextrins. It is also known that azobenzene-containing polymer is a widely applied functional monomer in photoirradiation of MIP. Therefore, ultraviolet–visible (UV-Vis) absorption spectra, fourier transform infrared spectroscopy (FTIR) spectra, and ^1^H-NMR were used to detect the properties of the 4-(Phenyldiazenyl)phenol (PDP) and MAA.

#### 2.1.1. Photoisomerization Properties of MAA

**Figure 1:**
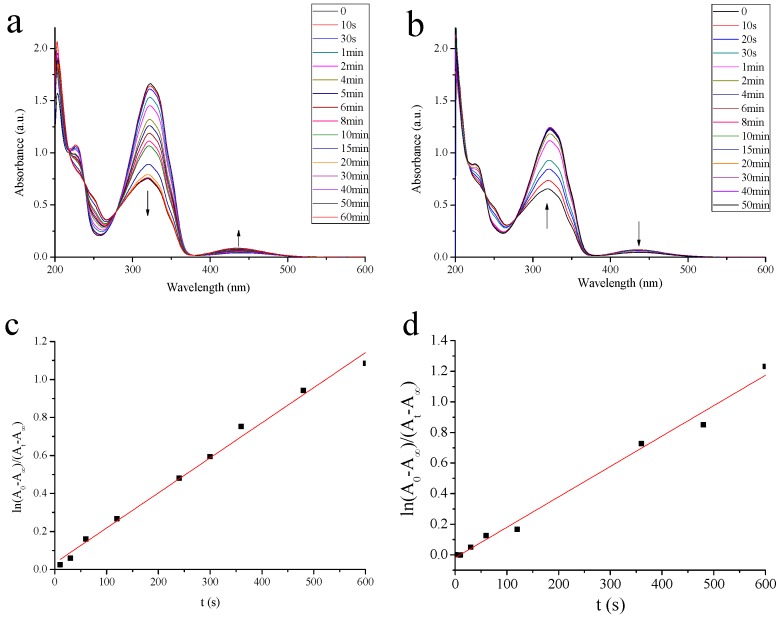
The ultraviolet–visible (UV-Vis) spectra and spectral changes of the 4-Methacryloyloxy Azobenzene (MAA). (**a**) Irradiation at 365 nm; (**b**) irradiation at 440 nm; (**c**) kinetics of the *trans* to *cis* photoisomerization; (**d**) kinetics of the *cis* to *trans* photoisomerization. a.u.: arbitrary units.

Figure 1 showed the spectroscopic responses of the MAA at room temperature upon alternate irradiation at 365 and 440 nm light. The MAA exhibited one strong absorption peak at ~326 nm and another very weak peak at ~440 nm, which are typical for the azobenzene chromophore and can be attributed to the π-π* and *n*-π* electron transitions of the N=N bond, respectively [19,24]. After irradiation with 365 nm light, the peak intensity at ~326 nm decreased rapidly, whereas the peak at ~440 nm increased over time (Figure 1a), which was attributed to the *trans* to *cis* photoisomerization of the azobenzene. Subsequent irradiation at 440 nm caused *cis* to *trans* photoisomerization (Figure 1b). However, the finally recovered absorbance of *trans*-MAA was lower than that previous irradiation at 365 nm light. Similar observations were reported by other studies [25,26]. The rate constants for the *trans* to *cis* and *cis* to *trans* photoisomerization were 2.54 × 10^−3^ s^−1^ and 11.9 × 10^−3^ s^−1^, respectively. Moreover, the photoisomerization properties became fully reversible upon subsequent cycles of the 365 nm and 440 nm irradiation (Figure S2 in the Supplementary Materials). No obvious decline of absorbance was found after five cycles. Similar phenomena were also observed previously by others [27,28]. It implied that the MAA has good restorability and reversibility in the process of UV and visible light irradiation. Similar results were also reported by Yang et al. [29], which used functional monomer 4-((4-methacryloyloxy)-phenylazo)benzenesulfonic acid to polymerize on the surface of silica microspheres.

#### 2.1.2. FTIR and ^1^H-NMR Spectroscopy of PDP and MAA

**Figure 2:**
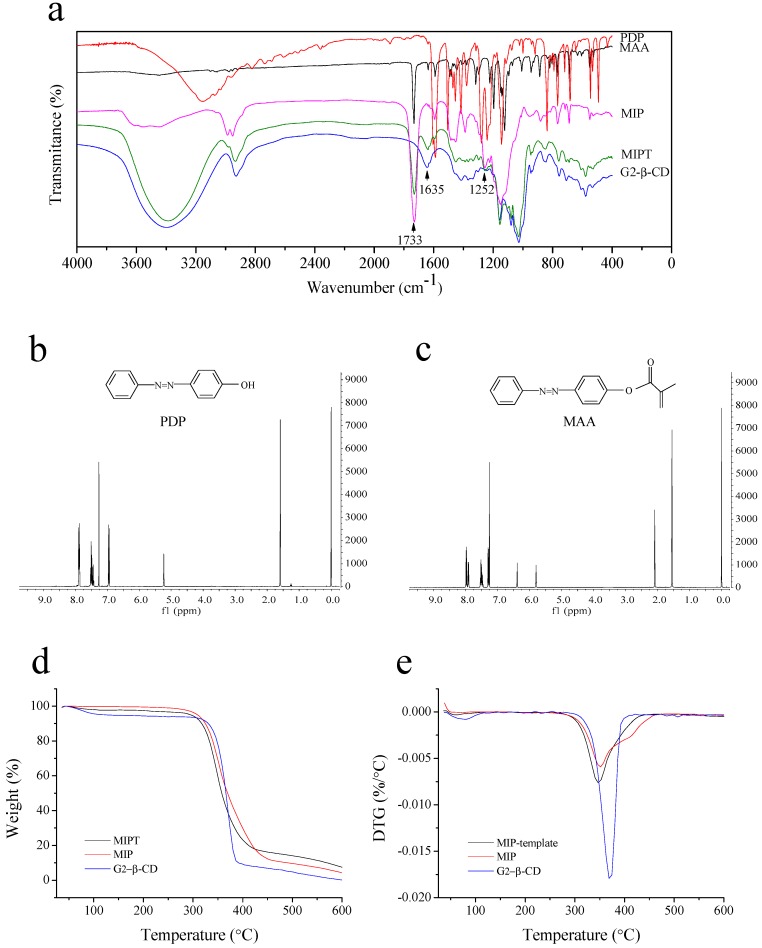
Fourier transform infrared spectroscopy (FTIR), thermogravimetric analysis (TGA) and ^1^H-NMR spectrum. (**a**) FTIR spectra; (**b**) ^1^H-NMR spectrum of 4-(Phenyldiazenyl)phenol (PDP); (**c**) ^1^H-NMR spectrum of MAA; (**d**) TGA thermograms; (**e**) derivative thermogravimetry (DTG) thermograms.

The FTIR spectra of the PDP and MAA were presented in Figure 2a. The characteristic peaks of PDP were located at 1488 cm^−1^ (υ_N=N_ azo group) and 1593 cm^−1^ (υ_C=C_ aromatic). The characteristic peaks of MAA were located at 2939 cm^−1^ (υ_CH_ aliphatic), 1737 cm^−1^ (υ_C=O_ methacrylic ester), 1644 cm^−1^ (υ_C=C_ methacrylic), and 1593 cm^−1^ (υ_C=C_ aromatic).

The ^1^H-NMR spectra of the PDP and MAA were shown in Figure 2b,c. The aromatic protons of PDP appeared between 6.95 and 7.88 ppm. The spectrum of MAA showed similar aromatic protons at 7.30–7.99 ppm. The three singlets at 2.09, 5.80, and 6.39 ppm were the methacrylic eater group and their signals were clearly detectable.

### 2.2. Characterization of MIPT and MIP

#### 2.2.1. Morphology of Samples

**Figure 3:**
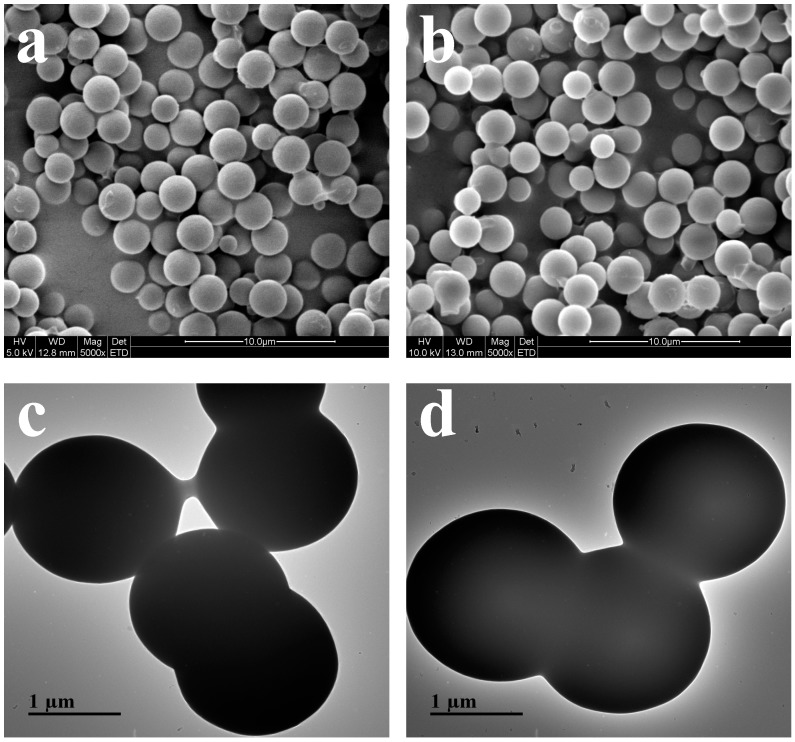
The scanning electron microscopy (SEM) and transmission electron microcopy (TEM) images. (**a**) SEM of the imprinted polymer containing the template (MIPT); (**b**) SEM of molecularly imprinted polymer (MIP); (**c**) TEM of MIPT; (**d**) TEM of MIP.

The scanning electron microscopy (SEM) images of MIPT (the Imprinted Polymer Containing the Template Form) and MIP were presented in Figure 3a,b. SEM revealed that the MIPT and MIP were uniform with a mean size of ~1.5–2 μm. The similar diameters of the MIPT and MIP revealed that G2-β-CD had less influence on the particle size, which agreed with the previous studies [27].

The size and morphology of the MIPT and MIP were characterized by transmission electron microcopy (TEM) (Figure 3c,d). TEM images showed that the MIPT and MIP were nearly spherical with size of ~2 μm. Figure 3d (TEM of MIP) showed a clear and direct visual evidence of the core-shell structure. More importantly, the TEM micrograph proved that the MIP has a core and a surface imprinting polymer shell, suggesting that G2-β-CD was removed from the MIP. Similar phenomena were also observed previously by others [28,30].

#### 2.2.2. FTIR Spectroscopy

The FTIR spectra of MAA, G2-β-CD, MIPT, and MIP were presented in Figure 2a. Three characteristic peaks of the existence of ethylene glycol dimethacrylate (EGDMA) attributed to the C=O stretching (1733 cm^−^^1^) and C–O–C stretching (1252 and 1144 cm^−^^1^) in the obtained MIPT and MIP could be observed. Furthermore, the presence of a well-defined peak of G2-β-CD at 1635 cm^−^^1^ (for H–O–H bending) [31,32,33] for MIPT suggested that G2-β-CD was imprinted into the MIPT. Compared with MIPT, weak absorption bands at 3402 cm^−1^ and the disappeared characteristic absorption at 1635 cm^−1^ could be observed in the spectra of MIP, indicating that G2-β-CD was removed from the MIP material.

#### 2.2.3. Thermogravimetric Analysis

Thermogravimetric Analysis (TGA) and derivative thermogravimetry (DTG) curves of G2-β-CD, MIPT, and MIP were presented in Figure 2d,e. There were two major weight loss in the TGA curves. The first weight loss occurred between 25 °C and 150 °C, and a second one occurred between 300 °C and 400 °C. In the first stage, the weight loss of G2-β-CD, MIPT, and MIP was 11.2%, 2.3%, and 0.3%, respectively. It may be due to the presence of certain compounds at low pyrolysis temperatures and the vaporization of adsorbed water. In the second stage, the weight of G2-β-CD, MIPT, and MIP reduced significantly to 9.4%, 30.9%, and 23.0%, respectively. The reduction of weight could be attributed to the oxidative decomposition of the most organic compounds and some volatile components. Finally, G2-β-CD, MIPT, and MIP retained ~0.1–8% solid carbonized residue (0.11%, 7.45%, and 4.28%, respectively). The DTG curves of all three samples showed a roughly consistent trend. However, there were several different transitions of the samples. Compared to MIP, the weight loss stage below 150 °C of MIPT was observed, which could be ascribed to the template contained in the MIPT. In DTG curve of G2-β-CD, similarly, the weight loss between 25 and 150 °C was observed. The results illustrated that G2-β-CD was imprinted into the MIPT and removed from the MIP material.

### 2.3. MIP and NIP Binding Properties

**Figure 4:**
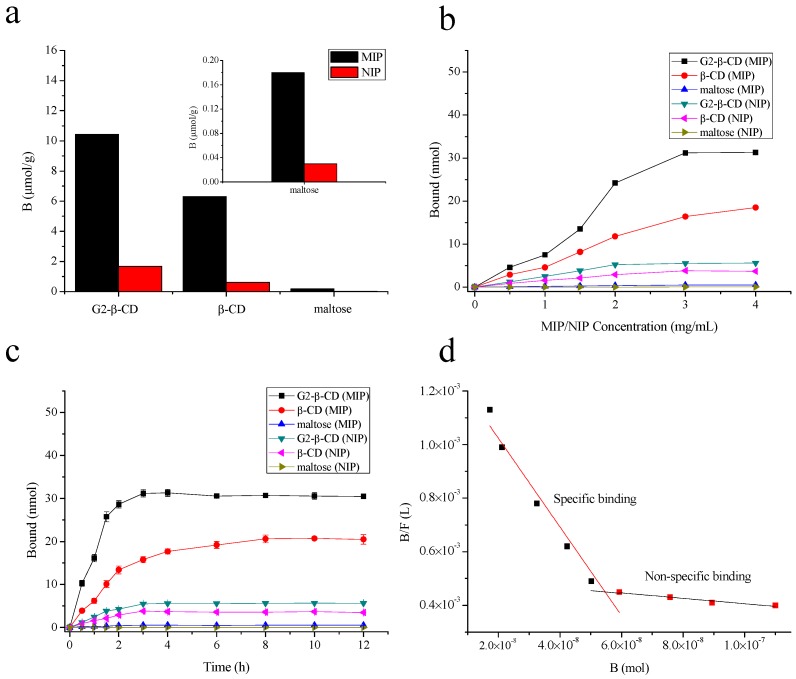
Binding properties of 6-*O*-α-d-maltosyl-β-cyclodextrin (G2-β-CD), β-cyclodextrin (β-CD), and maltose on MIP and molecularly non-imprinted polymer (NIP). (**a**) Binding selectivity; (**b**) equilibrium binding experiments; (**c**) binding isotherm; and (**d**) binding kinetics.

The selectivity of MIP toward G2-β-CD, β-CD, and maltose were presented in Figure 4a. The MIP had significantly lower binding capacities toward maltose and β-CD than G2-β-CD, thus suggesting a higher selectivity of MIP for G2-β-CD. For β-CD, the adsorption capacity was a little higher than maltose due to its extremely similar structure to G2-β-CD, which indicated that the recognition cavity of MIP was based on the shape, size, and functionality of the template.

The equilibrium binding experiments of the G2-β-CD with different amounts of MIP/NIP (molecularly non-imprinted polymer) were provided in Figure 4b. The binding amounts of G2-β-CD and β-CD increased rapidly with the concentration of MIP. Meanwhile, MIP has a higher binding capacity for G2-β-CD, β-CD, and maltose than NIP at the same concentration. Until the MIP concentration reached to 3 mg/mL, the binding capacity for G2-β-CD was achieved to equilibrium. However, there were little binding amounts of maltose while the MIP/NIP concentration increased. The possible reason was that MIP had generated selective recognition sites in the MIP cavities.

Figure 4c shows the binding kinetics of G2-β-CD, β-CD, and maltose on the MIP/NIP versus the different time. The binding amounts of G2-β-CD rapidly increased within 30 min, and equilibrium was achieved in 3 h with a binding capacity of ~62.6% (31.3 nmol). For the maltose, the binding capacity of MIP/NIP was nearly 0. Compared to MIP, NIP has a much lower adsorption capacity for G2-β-CD, β-CD, and maltose. The MIP exhibited much higher binding capacity and faster mass transfer rate for G2-β-CD because the existence of more effective binding sites allowed more G2-β-CD to bind rapidly [17].

The Scatchard analysis [34] of MIP with G2-β-CD was shown in Figure 4d. The two straight lines in the graph indicated that there were two different binding sites for MIP with G2-β-CD: non-specific binding and specific binding. The *K_a_* and *N*_max_ values were calculated and they were 1.72 × 10^4^ M^−1^ and the 7.93 μmol·g^−1^, respectively.

### 2.4. Photoregulated Release and Uptake of G2-β-CD and Maltose by the MIP

**Figure 5:**
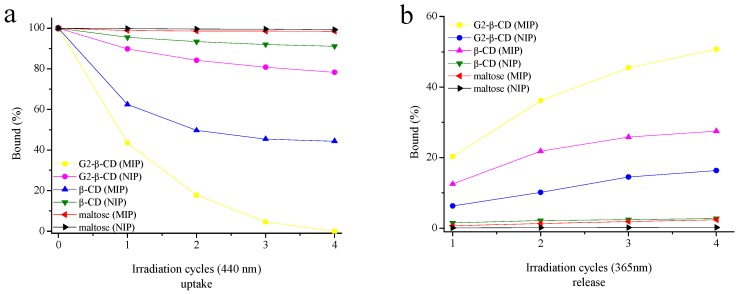
Photoregulated uptake and release of G2-β-CD, β-CD, and maltose by MIP and NIP. (**a**) photoregulated uptake of G2-β-CD, β-CD, and maltose by the MIPand NIP; (**b**) photoregulated releaseof G2-β-CD, β-CD, and maltose by the azocontaining MIP and NIP.

Figure 5 showed the changes in the binding amount of crude G2-β-CD in the presence of MIP or NIP under alternate irradiation at 365 and 440 nm light. UV light (365 nm) irradiation caused the *cis*-azobenzene to return to *trans*-azobenzene [35,36], which was unable to form inclusion complexes with CDs, leading to the release of CDs from MIP into the solution. Then the subsequent irradiation at 440 nm caused MIP rebound CDs. The MIP has significantly higher adsorption capacity for G2-β-CD, β-CD, and maltose than NIP. After the suspension was incubated in a dark environment, the adsorption rates of G2-β-CD by MIP and NIP were 56.48% and 10.2%, respectively. The MIP and NIP towards β-CD showed a binding of 37.51% and 6.5%, respectively. The subsequent irradiation at 365 nm led to the release of G2-β-CD and β-CD from MIP (20.3% and 12.5%, respectively) and NIP (6.3% and 1.5%, respectively) into solution. The subsequent irradiation at 440 nm caused a rebound in the G2-β-CD and β-CD binding. When repeating irradiation cycles, almost all of G2-β-CD were rebounded from the solution into the MIP with four cycles and 50.76% of G2-β-CD was released from the MIP into the solution with four cycles. The obtained G2-β-CD was separated and purified by MIP with the purity of 90.8% (Figure S1e in the supporting information). The change of the amount of uptake and release of β-CD was 44.39% and 27.52% under irradiation of MIP with 365 and 440 nm light with four cycles. However, both the MIP and NIP showed lower release and uptake ability of maltose.

## 3. Materials and Methods

### 3.1. Materials

*N*,*N*-Dimethylaminopyridine (DMAP), 2,2-Azobisisobutyronitrile (AIBN), Ethylene glycol dimethacrylate (EGDMA, 98%), tetramethylsilane (TMS), and triethylamine (TEA) were purchased from Sigma-Aldrich (St. Louis., MO, USA). G2-β-CD was purchased from Sinopharm Chemical Regent Co., Ltd. (Shanghai, China). All other chemicals consumptions were at the analytical level.

### 3.2. Synthesis of 4-(Phenyldiazenyl)phenol

4-(Phenyldiazenyl)phenol was prepared following a previously reported procedure [37] (Scheme S1 in the Supplementary Materials). The solution of concentrated HCl (8 mL) and water (8 mL) was cooled to 0 °C and aniline (2.51 g, 27 mmol) was dropwise added. Then, NaNO_2_ (2.00 g, 29 mmol) in water (10 mL) was slowly added into the precooled solution. Next, the solution was constantly stirred in an ice bath for 25 min. After the mixture, separately, phenol (2.54 g, 27 mmol) was dissolved in 25 mL of 10% NaOH solution. The prepared solution was slowly added into the diazonium salt solution with stirring while the temperature was maintained at 5 °C for 50 min. The yellow-orange pellet was collected by filtration and washed by water. The crude solid was recrystallized with an ethanol/water mixture (yield 74.5%).

### 3.3. Synthesis of 4-Methacryloyloxy Azobenzene 

**Scheme 1:**
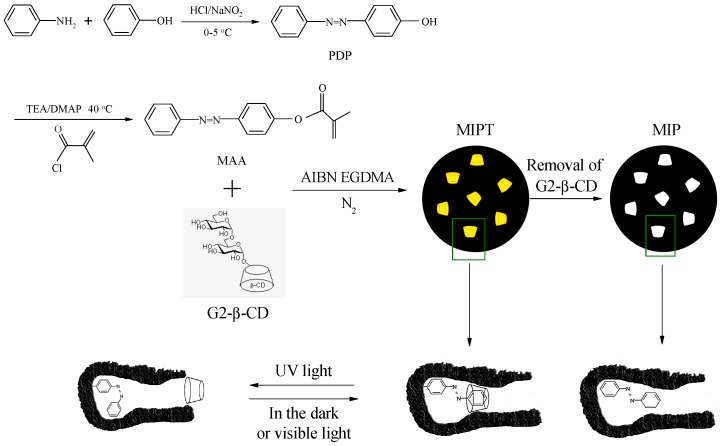
Synthetic route for the photoirradiation molecular imprinting polymer. TEA: triethylamine; DMAP: *N*,*N*-Dimethylaminopyridine; AIBN: 2,2-Azobisisobutyronitrile; EGDMA: ethylene glycol dimethacrylate.

The functional monomer MAA was synthesized according to a previously reported method [19] with some modifications (Scheme 1). DMAP (0.15 g, 1.0 mmol), TEA (4.30 g, 42 mmol), and PDP (3.96 g, 20 mmol) were dissolved in a 150 mL of anhydrous acetonitrile and cooled over an ice bath, resulting in a dark red mixture. After methacrylic chloride (4.50 g, 40 mmol) was added dropwise, the solution was stirred at 40 °C for 24 h and then cooled to room temperature. After saturated brine solution (50 mL) was added, the formed precipitate was collected by filtration, washed by 2 mol/L HCl, and dried with freeze-drying. The purified MAA was collected by recrystallization from boiling glacial acetic acid, yielding 61.7%.

### 3.4. Preparation of the MIP

The MIP was prepared according to a previously method [19] with some modifications. MAA (0.3990 g, 1.5 mmol), G2-β-CD (2.1889 g, 1.5 mmol), and anhydrous acetonitrile (75 mL) were added to a conical flask (100 mL). The mixture was stirred at room temperature for 4 h in a dark environment. Then, EGDMA (0.85 mL, 4.5 mmol) and AIBN (0.0086 g, 0.0525 mmol) were added into the above system. The reaction mixture was bubbled with nitrogen in an ice bath for 30 min, then the conical flask was sealed and dipped into a 60 °C oil bath. After 48 h of polymerization in a dark environment, the resultant polymer (MIPT, the imprinted polymer containing the template form) was collected by centrifugation. The G2-β-CD was removed from the MIPT through Soxhlet extraction with water at 110 °C for 48 h. The resultant material was dried at 35 °C under vacuum for 24 h to obtain MIP. As a control, molecularly non-imprinted polymer was prepared in the absence of G2-β-CD and treated with the same method.

### 3.5. Characterization of PDP and MAA

^1^H-NMR (400 MHz) was measured by a Bruker Advance III instrument (Bruker, Rheinstetten, Germany) at room temperature using TMS as an internal standard.

The FTIR spectra of the samples were recorded by an FTIR spectrometer (470 FTIR, Nicolet, Waltham, MA, USA). The dried samples were mixed with KBr, grounded, and pressed into a pellet. FTIR spectra were obtained in the wave number range from 400 to 4000 cm^−1^.

### 3.6. Photoisomerization Studies

A UV–Vis scanning spectrophotometer (Shimadzu-2600, Osaka, Japan) was used to record the UV–Vis spectra of MAA. The photoisomerization of the MAA was investigated by first irradiating with 365 nm light and then with 440 nm light. The photoisomerization kinetics was analyzed by the following equation:

(1) $$\ln\frac{A_{0}-A_{\infty }}{A_{t}-A_{\infty }}=kt$$

where *A*_0_, *A_t_*, and *A_∞_* are the absorbance of the azobenzene chromophores at their correspondence wavelengths at time 0, *t*, and at the photostationary stage, respectively; *k* is the rate constant of the photoisomerization process.

### 3.7. Scanning Electron Microscopy and Transmission Electron Microscopy

The morphology of the MIPT and MIP were observed by a SEM microscope (S-4800, Hitachi, Tokyo, Japan) at an accelerating voltage of 10 kV. The samples were coated with 10-nm-thick platinum in the aim of conductivity.

MIPT and MIP images were examined using a TEM microscope (HT-7700, Hitachi Instruments Ltd., Tokyo, Japan). The powdered MIPT and MIP were diluted and spread onto the copper grids coated with a carbon-supported film. The copper grids were left aside to stand for freeze-drying.

### 3.8. Fourier Transform Infrared Spectroscopy

FTIR spectra of G2-β-CD, MIPT, and MIP were recorded by an FTIR spectrometer (470 FTIR, Nicolet, Waltham, MA, USA). The dried samples were mixed with KBr, grounded, and pressed into a pellet. FTIR spectra were obtained in the wave range from 400 to 4000 cm^−1^.

### 3.9. Thermogravimetric Analysis

The thermogravimetric curves of G2-β-CD, MIPT, and MIP were performed by a TGA/SDTA851e (Mettler, Toledo, USA). The samples (4.0 ± 1.0 mg) were heated from 25 to 600 °C with a platinum crucible under air atmosphere at a flow rate of 20 mL/min. The heating rate was 10 °C/min.

### 3.10. Binding Selectivity

We dispersed 3.0 mg of MIP/NIP in the 1.0 mL of 5.0 × 10^−5^ mol/L G2-β-CD, β-CD and maltose, respectively. The suspension was shaken at 25 °C in a dark environment for 24 h and filtered using a 0.22 mm porous membrane. The concentration of G2-β-CD and maltose in the supernatant was measured by a high-performance liquid chromatograph (Shimadzu, Kyoto, Japan). The HPLC condition was as follows: Hypersil NH_2_ column (4.6 mm diameter × 250 mm), K-2301 refractive index detector (RID), mobile phase of acetonitrile—water (70:30 *v*/*v*), flow rate of 1 mL/min, temperature of 30 °C, injection of 10 μL.

### 3.11. Equilibrium Binding Experiments

We incubated 5.0 × 10^−5^ mol/L of G2-β-CD or maltose and different amounts of the MIP/NIP at 25 °C in a dark environment for 6 h. The amounts of G2-β-CD and maltose which bound to the MIP were then measured by HPLC.

### 3.12. Binding Kinetics

We incubated 3.0 mg of MIP/NIP and 1.0 mL of 5.0 × 10^−5^ mol/L G2-β-CD or maltose in a dark environment at 25 °C [38]. After different intervals, the suspensions were sampled and filtered using a 0.22 mm porous membrane. The adsorption dynamics were tested by detecting the amounts of G2-β-CD and maltose remaining in the solution. The concentration of free G2-β-CD and maltose was measured by HPLC.

### 3.13. Binding Isotherm

We incubated 3 mg of MIP/NIP and a series of G2-β-CD solutions (C = 1.0 × 10^−5^–1.5 × 10^−4^ mol/L, 1.0 mL) at 25 °C for 6 h. The suspensions were filtered using a 0.22 mm porous membrane, and then the amounts of G2-β-CD bound to the MIP (*B*) were measured by HPLC. The equilibrium adsorption capacity of G2-β-CD by the MIP was calculated through the Scatchard equation:

(2) $$\frac{B}{F}=(N_{\max}-B)K_{a}$$

where *F* is the amount of free G2-β-CD in the solution, *K_a_* is the binding association constant and *N*_max_ is the maximum number of the binding site.

### 3.14. Photocontrolled Uptake and Release

The G2-β-CD was produced through the reverse action with pullulanase in a reaction mixture containing of β-CD and maltose. The reaction mixture was analyzed by HPLC and five peaks were observed (Figure S1b in the Supplementary Materials). The β-CD has a structure similar to G2-β-CD, it has great significance to separate β-CD from the obtained crude G2-β-CD by 50% methanol owing to the different solubility of productions [39]. The β-CD could be precipitated from the system easily as precipitation, and the recovery ratio was around 60% (Figure S1c in the Supplementary Materials). The further purification of G2-β-CD was performed with MIP. The photocontrolled release and uptake studies of the crude G2-β-CD by the MIP/NIP were performed by alternate irradiation under 365 and 440 nm light. 3.0 mg MIP/NIP was dispersed in the 1.0 mL of 0.5 mg/mL crude G2-β-CD solution. After the suspension was incubated at 25 °C in a dark environment for 6 h, the samples were centrifuged to determine the amounts of G2-β-CD, β-CD, and maltose in the supernatant by HPLC. The precipitate obtained by the centrifugation was dispersed in 1.0 mL of distilled water with stirring and irradiated at 365 nm light for 4 h. Then, stirring was stopped and the centrifugation was performed again. The amounts of G2-β-CD, β-CD, and maltose in the supernatant were measured by HPLC. The precipitate of centrifugation and the supernatant of the first centrifugation conducted were stirred in the visible light (440 nm). After 6 h of visible light irradiation at 25 °C, the samples were centrifuged to determine their binding. The photocontrolled release and uptake studies (i.e., UV light on for 4 h and visible light on for 6 h, alternately) were conducted similarly as above. The amounts of samples in the supernatant were determined as well.

### 3.15. Statistical Analysis

The data were represented as the averaging of at least three replications. The data were statistically analyzed using SPSS 17.0 (SPSS Inc., Chicago, IL, USA). The experimental data were analyzed using analysis of variance (ANOVA) by the Origin Pro 8.0 statistics program (Originlab, Northampton, MA, USA) and represented by mean ± standard deviation. The statistically significant differences were set at a significance level of 95% (*p* < 0.05).

## 4. Conclusions

The innovative usage of molecular imprinting technology, which is an environmentally friendly method, for the selective separation of the branched CDs resulted in a good performance at an affordable cost, thus this process is potentially suitable for lab scale. The MIP was prepared using G2-β-CD as the template. The MIP was used to isolate G2-β-CD from the reaction mixture. The photoirradiation MIP had a core-shell structure for the photocontrolled uptake and release of G2-β-CD. FTIR and TGA showed that G2-β-CD was imprinted into the polymer and removed from MIP. With alternate irradiation at 365 and 440 nm light, MIP reversibly released and rebound to the G2-β-CD, which resulted in the amount of G2-β-CD in the solution being nearly 0. The presented method was sensitive, effective, and simple. Therefore, it could be used for the selective separation of the branched CDs. MIP was an effective method of isolation and purification branched CDs for getting enough G2-β-CD to gain the industrial preparation and application.

## Supplementary Materials

Supplementary materials are available online.
