# Supplementary material for: Preparation of Photoirradiation Molecular Imprinting Polymer for Selective Separation of Branched Cyclodextrins
Source: Molecules. 2017 Feb 21;22(2):288. doi: 10.3390/molecules22020288 (PMC6155763; doi:10.3390/molecules22020288)
Supplement: Supplementary file 1 [file molecules-22-00288-s001.pdf]

# Supplementary Materials: Preparation of Photoirradiation Molecular Imprinting Polymer for Selective Separation of Branched Cyclodextrins

**Haoran Fan, Jinpeng Wang, Qingran Meng, Xueming Xu, Tianming Fan and Zhengyu Jin**

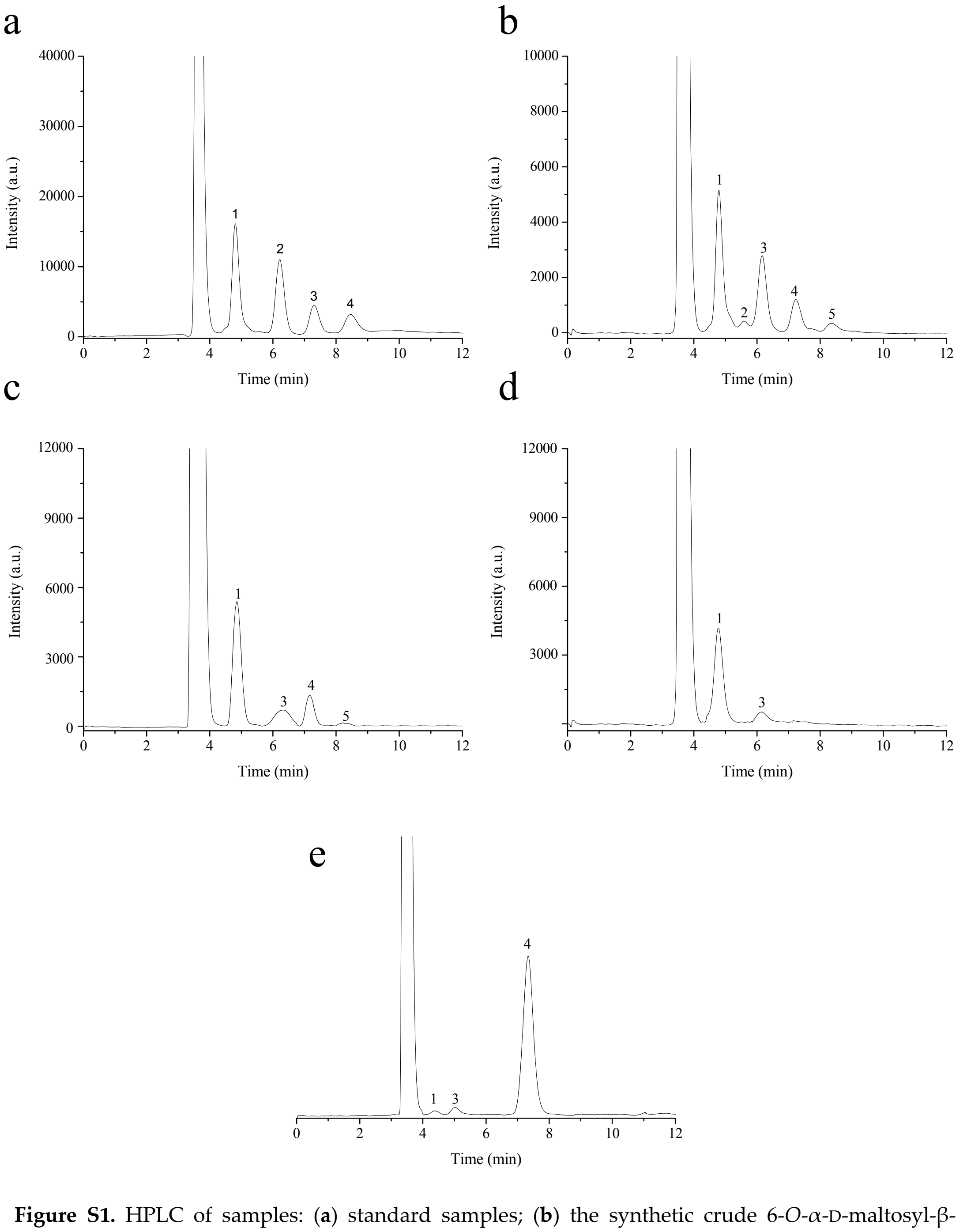


**Figure S1.** HPLC of samples: (**a**) standard samples; (**b**) the synthetic crude 6-*O*-α-D-maltosyl-β-cyclodextrin (G2-β-CD); (**c**) the synthetic crude G2-β-CD in 50% methanol; (**d**) the precipitation after irradiation under 365 nm light; (**e**) the separated and purified G2-β-CD by molecularly imprinted polymer (MIP). Peaks: 1. maltose; 2. maltosyl maltose; 3. β-cyclodextrin; 4. maltosyl β-cyclodextrin; 5. dimaltosyl β-cyclodextrin. a.u.: arbitrary units.

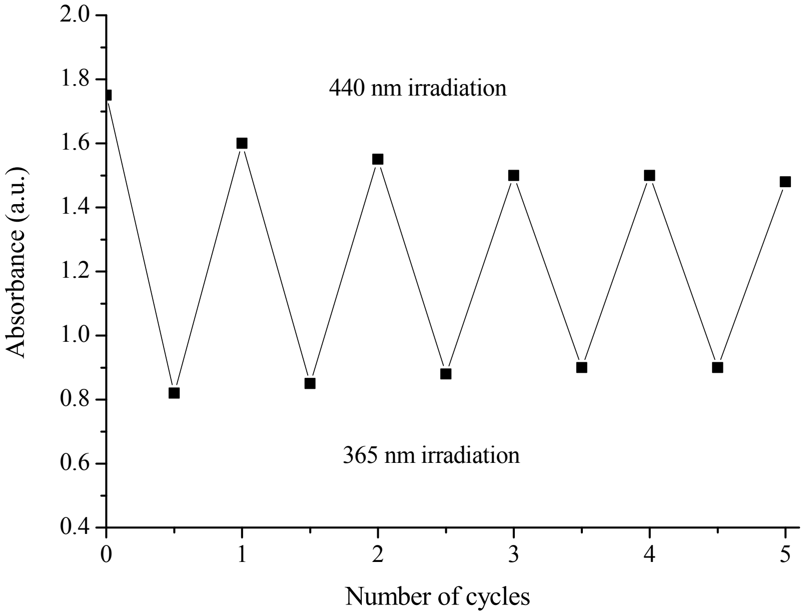


**Figure S2.** Reversibility of the photoisomerization process of azobenzene chromophore in MIP at 25 °C. In each cycle, the azo monomer solution was irradiated firstly with 365 nm ultraviolet (UV) light for 50 min and then with 440 nm visible light for 10 min, respectively. a.u.: arbitrary units.
